# GRB7-mediated enhancement of cell malignant characteristics induced by *Helicobacter pylori* infection

**DOI:** 10.3389/fmicb.2024.1469953

**Published:** 2024-09-18

**Authors:** Huilin Zhao, Si Chen, Xinfeng Bai, Jianhui Zhang, Shuzhen Liu, Zekun Sun, Xinying Cao, Jianping Wang, Ying Zhang, Boqing Li, Xiaofei Ji

**Affiliations:** ^1^Department of Pathogenic Biology, School of Basic Medical Sciences, Binzhou Medical University, Yantai, China; ^2^Xu Rongxiang Regenerative Medicine Research Center, Binzhou Medical University, Yantai, China; ^3^Department of Laboratory Medicine, The Second People’s Hospital of Lianyungang, Lianyungang, China; ^4^Translational Medicine Research Center, Shandong Provincial Third Hospital, Shandong University, Jinan, China; ^5^The Second School of Clinical Medicine, Binzhou Medical University, Yantai, China

**Keywords:** *Helicobacter pylori*, GRB7, gastric cancer, STAT3, CagA

## Abstract

Growth factor receptor bound protein 7 (GRB7) is reportedly upregulated in human gastric cancer (GC), which is closely associated with tumor progression and prognosis. However, the mechanism underlying its dysregulation in GC remains poorly understood. In this study, we found that GRB7 overexpression was associated with *Helicobacter pylori* (*H. pylori*) infection. GC cells (AGS and MGC-803) infection assays revealed that this upregulation was mediated by the transcription factor STAT3, and activation of STAT3 by *H. pylori* promoted GRB7 expression in infected GC cells. Moreover, CagA, the key virulence factor of *H. pylori*, was found involved in STAT3-mediated GRB7 overexpression. The overexpressed GRB7 further promoted GC cell proliferation, migration, and invasion by activating ERK signaling. Mice infection was further used to investigate the action of GRB7. In *H. pylori* infection, GRB7 expression in mice gastric mucosa was elevated, and higher STAT3 and ERK activation were also detected. These results revealed GRB7-mediated pathogenesis in *H. pylori* infection, in which *H. pylori* activates STAT3, leading to increased GRB7 expression, then promotes activation of the ERK signal, and finally enhances malignant properties of infected cells. Our findings elucidate the role of GRB7 in *H. pylori*-induced gastric disorders, offering new prospects for the treatment and prevention of *H. pylori*-associated gastric carcinogenesis by targeting GRB7.

## Introduction

Gastric cancer (GC) is the fourth leading cause of cancer-related deaths worldwide (Sung et al., 2021). The majority of GC patients (>80%) are diagnosed at advanced stages due to its asymptomatic nature. Despite advances in surgical and chemotherapeutic treatments, the 5-year survival for GC patients remains below 30% (Thrift and El-Serag, 2020; Joshi and Badgwell, 2021; Allemani et al., 2015). Therefore, investigation of the mechanisms underlying GC development, specifically by exploring effective specific markers and potential therapeutic targets to enable early diagnosis and treatment, is urgently required.

*Helicobacter pylori* (*H. pylori*) infection is a high-risk factor for the occurrence and progression of GC (Correa, 1992; Montecucco and Rappuoli, 2001; Amieva and Peek, 2016). Carcinogenic mechanisms associated with *H. pylori* are multifactorial, including bacterial virulence, host immune responses and environmental factors (Wroblewski and Peek, 2023). A series of *H. pylori* virulence factors contribute to the disease progression, such as type IV secretion system (T4SS), cytotoxin-associated gene A (CagA), vacuolating cytotoxin A (VacA), serine protease HtrA, and the outer-membrane adhesins (Wu et al., 2013; Cover and Blaser, 2009; Yang et al., 2018). Among these virulence factors, CagA is regarded to has the direct oncogenic action (Hatakeyama, 2023). After delivered into gastric epithelial cells via T4SS, CagA can interact with a variety of signaling factors to hijack intracellular signal transduction pathways (Franco et al., 2005; Tabassam et al., 2009; Lee et al., 2010; Bronte-Tinkew et al., 2009; Polk and Peek, 2010), and further trigger the progress of carcinogenesis. Additionally, *H. pylori* induces chromosomal DNA damage closely connected with CagA (Backert et al., 2023).

Growth factor receptor bound protein 7 (GRB7) is a member of the signal transduction protein family, which includes GRB7, GRB10, and GRB14. Structurally, GRB7 comprises an N-terminal proline-rich (PR) region, a GM (Grb and Mig) region, and a C-terminal SH2 domain. The N-terminal PR region is responsible for SH3-mediated protein–protein interactions (Han et al., 2001). The carboxyl SH2 domain plays a vital role in the interaction of GRB7 with receptor tyrosine kinases and other tyrosine-phosphorylated signaling molecules (Thommes et al., 1999; Janes et al., 1997; Keegan and Cooper, 1996). As an adaptor-type signaling protein, GRB7 can act as a transducer for stimulating growth factor-like downstream signals, resulting in cell morphological changes and proliferation (Han et al., 2001; Chu et al., 2019). Moreover, GRB7 can transduce multiple signaling pathways by regulating the phosphorylation of AKT, ERK1/2, and paxillin (Wang et al., 2010; Zheng et al., 2020; Wang et al., 2020; Paudyal et al., 2013; Pero et al., 2002).

GRB7 has been found to amplify oncogenic signals, promoting cancer development (Zheng et al., 2020; Wang et al., 2020; Paudyal et al., 2013; Pero et al., 2002; Gotovac et al., 2020; Zhao et al., 2017; Chu et al., 2010; Pero et al., 2003; Pei et al., 2022; Zhu et al., 2023). For example, GRB7 overexpression has been linked to increased tumor proliferation and migration in cervical and ovarian cancers (Wang et al., 2010; Zhao et al., 2017) and enhanced cell invasion in breast cancer and esophageal adenocarcinoma (Gotovac et al., 2020; Chu et al., 2010). Furthermore, clinical statistics have demonstrated that GRB7 overexpression is associated with cancer recurrence and a worse prognosis in these cancer patients (Pero et al., 2003). Silencing GRB7 in cell experiments and mouse tumor models has led to reduced proliferation and tumorigenesis in bladder cancer (Zheng et al., 2020). These findings underscore GRB7 as an attractive target for therapeutic intervention. Recently, the association between GRB7 overexpression and GC development has been delved (Pei et al., 2022; Zhu et al., 2023). Our study also found the upregulation of GRB7 in GC and its negative correlation with survival time. Moreover, Selbach et al. (2009) reported the interaction of GRB7 and intracellular phosphorylated CagA. These findings set us thinking the action of GRB7 in gastric carcinogenesis and whether it is involved in *H. pylori* pathogenesis.

In this study, we revealed the association between GRB7 expression and *H. pylori* infection. Thereafter, we investigated the regulation of GRB7 expression by *H. pylori* and profiled the role of GRB7 in promoting gastric carcinogenesis and its potential involvement in *H. pylori* pathogenesis.

## Materials and methods

### Cell lines and GC tissues

The GC cell lines (MGC-803, MKN-45, AGS, SGC-7901) and the human immortalized gastric epithelial cell line (GES-1) were used in this study. MGC-803 and GES-1 cells were cultured in DMEM medium (Macgene, Beijing, China) with 10% (v/v) FBS (Gibco, Grand Island, United States). While cells of MKN-45, AGS and SGC-7901 were cultured in RPMI 1640 (Procell, Wuhan, China) medium with 10% (v/v) FBS (Gibco). All the cells were cultured at 37°C in a humidified environment containing 5% CO_2_. GC tissues and paracancerous tissues were supplied by Yantai Affiliated Hospital of Binzhou Medical University, which was approved with written informed consent.

### *Helicobacter pylori* cultivation and infection

*Helicobacter pylori* strains 26,695 and SS1 were used in this study, in which 26,695 strain was used in cell infection assays and SS1 strain was used in mice infection. CagA deleted *H. pylori* 26,695 strain (CagA^−^) was constructed in previous studies by homologous recombination as described by Ji et al. (2016) *H. pylori* cells were cultivated in Kamani (OXOID, Beijing, China) plates supplemented with 5% sterile defibrinated sheep blood under a microaerobic environment at 37°C. In cell infection assays, cells of 26,695 strain were collected and suspended in sterile PBS and added into cell culture at a 100:1 multiplicity for 4 h of infection. For mice infection, SS1 cells were suspended in brain heart infusion broth and inoculated orally.

### Immunohistochemical staining

Immunohistochemical (IHC) staining was used to analysis the expression levels of GRB7, E-cadherin and Ki67 in gastric mucosal tissue samples. IHC staining was performed according to the usual procedure by using IHC test reagents (ZSGB-BIO, Beijing, China) (Song et al., 2004). The slides were sequentially processed by dewaxing, hydration, incubation with primary antibody [Rabbit polyclonal anti-GRB7 (Proteintech, Wuhan, China), E-cadherin (Affinity, Jiangsu, China), Ki67 (Proteintech, Wuhan, China)] at 4°C overnight, and treatment with HRP-labeled anti-rabbit antibody (ZSGB-BIO, Beijing, China) for 1 h at 37°C. DAB chromogenic solution was then added dropwise onto the slides. Counterstaining of the cell nuclei was performed by using hematoxylin. The expression level was scored by two independent observers who were blinded to the clinical outcomes. The studies involving human participants were reviewed and approved by Ethics Committee of Binzhou Medical University. The patients/participants provided their written informed consent to participate in this study.

### Histological analysis

Hematoxylin-eosin (HE) staining was preformed to assess pathological changes of gastric mucosal epithelium in *H. pylori* infection. It was carried out follow the usual steps (Liao et al., 2024). Briefly, the stomach tissues collected from mouse models were fixed in 4% paraformaldehyde overnight and embedded in paraffin. Then, sections with a thickness of 4 μm were cut from the block and stained with hematoxylin-eosin (HE) or Giemsa dye solution.

### Cell transfection

To downregulate GRB7, NF-κB, β-catenin, c-Jun, and STAT3, we transfected corresponding small interfering RNA (siRNA; Tsingke, Beijing, China) into cells using Lipofectamine RNAiMAX (Invitrogen, Grand Island, United States). For overexpression, genes *GRB7* and *STAT3* were synthesized (Tsingke, Beijing, China) and separately cloned into the pcDNA3.1 vector (Invitrogen). The resulting plasmids (pcDNA3.1-GRB7 and pcDNA3.1-STAT3) were introduced into cells using Lipofectamine 2000 (Invitrogen) according to the manufacturer’s instructions. Transfection efficiency was assessed through western blot analysis. The target siRNA sequences are listed in Supplementary Table S1.

### Western blot analysis

Cells were lysed in RIPA buffer (Solarbio, Beijing, China) supplemented with Phenylmethanesulfonyl fluoride (Beyotime, Shanghai, China) on ice. The protein concentration was estimated by BCA Protein Assay Kit (Beyotime). The same quantity of proteins was separated in SDS-PAGE and transferred to a PVDF membrane, which was then blocked with 5% nonfat milk for 2 h at 25°C, and incubated with the primary antibody overnight at 4°C. After washing off the primary antibody, the membrane was incubated with a horseradish peroxidase-conjugated secondary antibody for 1 h at 25°C. Finally, the membrane was soaked in ECL Western Blotting Substrate (Novland BioPharma, Shanghai, China) and the protein bands were colored and assessed using an enhanced chemiluminescence system (Tanon, 5200, China). Antibodies used in this study are as follows: anti-GRB7, p-MEK, MEK, ERK, STAT3 and p-STAT3 were purchased from ABclonal (Wuhan, China); anti-β-actin, CDK6, Cyclin D1, p27, p-ERK and GAPDH were obtained from Proteintech (Wuhan, China); anti-E-cadherin and Vimentin were provided by Affinity (Jiangsu, China); anti-CagA were purchased from Santa Cruz Biotechnology (Texas, United States).

### Real-time quantitative PCR

Real-time quantitative PCR (RT-qPCR) was performed to detect transcription levels of *GRB7* gene in *H. pylori* infection. Total RNA was extracted from cells using TRIzol reagent (Invitrogen), and SPARKscript II RT Plus Kit (Sparkjade, Qingdao, China) was chosen to reverse-transcribes RNA to cDNA. To assess the intracellular GRB7 mRNA levels, RT-qPCR was performed using SYBR Green reagent (Sparkjade). The expression data were normalized to the geometric mean of the housekeeping gene GAPDH and analyzed with the 2^−ΔΔCt^ method (Livak and Schmittgen, 2001). The primer sequences adopted are as follows: GRB7 (5′-GGTTTGGAGGACCACGAGTC-3′, 5′-CGGAAGACGAAGCGGCTATC-3′), GAPDH (5′-GTATCGTGGAAGGACTCATGAC-3′, 5′-ACCACCTTCTTGATGTCATCAT-3′).

### Colony formation assays

Colony formation assay was used to analyze the effect of GRB7 on cell proliferation, which was performed as Lei et al. (2017) described. Briefly, AGS or MGC-803 cells were seeded in six-well plates at a density of 500 cells/well. After incubation for 10–14 days, the cells were fixed with 4% paraformaldehyde and stained with 0.1% crystal violet. Visible colonies were counted under microscope (Olympus, N2179000, Japan).

### Cell migration and invasion assay

Transwell assays were carried out to investigate the effects of *H. pylori* infection and GRB7 expression changes on cell motility (Zhu et al., 2020). For migration and invasion experiments, 24-well Transwell plates with 8 μm pore size (Corning, New York, United States) were used. The test AGS and MGC-803 cells, suspended in 200 μL serum-free medium (Gibco), were seeded into the upper chamber. The lower chamber was filled with 600 μL medium containing 20% FBS, serving as a chemoattractant. For invasion tests, Matrigel (Abwbio, Shanghai, China) was spread on the basement membrane of the upper chamber before seeding. After 24 h of cultivation, cells on the lower surface of the chamber were fixed using 4% paraformaldehyde, stained with 0.1% crystal violet, and counted under a microscope (Olympus).

### Cell apoptosis assay

Cell apoptosis assay was used to analysis the effect of GRB7 on the apoptosis rate of AGS and MGC-803 cells. Apoptosis test was performed as general procedures (Wang et al., 2021) by using the Annexin V-FITC/propidium iodide (PI) kit (Solarbio). Briefly, AGS and MGC-803 cells were digested with EDTA-free trypsin (Solarbio) to prepare cell suspensions. Following a single wash with cold PBS, cell suspensions were consecutively stained with 5 μL of Annexin V and PI in the dark for 5 min. Finally, the cell suspensions were analyzed using flow cytometry (BD Biosciences, BD Canto II, United States).

### Cell cycle assay

Cell cycle assay was performed by using flow cytometry to analysis the effect of GRB7 on the cell cycle, which was proceeded as Li et al. (2021) described. Briefly, the test AGS and MGC-803 cells were initially incubated in 70% pre-cooled ethanol overnight at 4°C and treated with 100 μL of RNase A (Solarbio) at 37°C for 30 min. After adding 400 μL of PI stain (Solarbio), cells were incubated for an additional 30 min in the dark at 4°C. The distribution of cells in different phases of the cell cycle was analyzed using flow cytometry (BD Biosciences).

### Wound-healing assay

Wound-healing assays were used to analyze the effect of GRB7 on cells migration (Grada et al., 2017). AGS and MGC-803 cells were seeded in a six-well plate and cultured until confluence was reached to 80%. To establish wounding, a 10 μL pipette tip was used to make a vertical and gentle line across the center of the well. The detached cells were rinsed off by washing with PBS. After cultivation for 12 h, the cell’s migration was observed by using a microscope (Olympus).

### Enzyme-linked immunosorbent assay

The secrtion of IL6 was measured with the IL6 enzyme-linked immunosorbent assay (ELISA) kit (mlbio, Shanghai, China), according to the manufacturer’s instructions.

### Immunofluorescence

Immunofluorescence was used to characterize the expression of GRB7 protein in AGS and MGC-803 cells, which was proceeded as Im et al. (2019) described. Culture cells were fixed on coverslips with ice-cold 4% paraformaldehyde for 30 min and permeabilized with 0.3% Triton X-100 for 15 min. After blocking with 5% goat serum at 37°C for 1 h, the samples were firstly incubated with primary antibodies (proteintech) at 4°C overnight, and then incubated with fluorescently labeled secondary antibodies (ABclonal) in dark at 25°C for 2 h. After washed with PBS, the samples were stained with DAPI (Beyotime). Finally, the samples were visualized and taken by Zeiss laser confocal scanning microscope (LSM880, German).

### 5-Ethynyl-2′-deoxyuridine staining

5-Ethynyl-2′-deoxyuridine (EdU) staining was used to analyze the effect of GRB7 expression changes on cell proliferation and DNA synthesis (Buck et al., 2008). BeyoClick^™^ EdU-555 Cell Proliferation Assay Kit (Beyotime) was used in this test. Briefly, AGS and MGC-803 cells were incubated with a pre-warmed working solution of EdU (10 μM) at 25°C for 2 h and fixed with 4% paraformaldehyde for 15 min. After washed three times with a washing solution, cells were incubated with permeabilization solution for another 15 min at 25°C and washed twice with the washing solution again. Nuclei were stained by 1× Hoechst 33342 solution for 10 min at 25°C in the dark. Zeiss laser confocal scanning microscope was used to detect fluorescence.

### Animal infection assay

To construct a mouse model of *H. pylori* colonization infection. Male mice on C57BL/6 background aged 6 weeks were purchased from Jinan Pengyue Laboratory Animal Breeding Co. (China). Before infection, the mice were maintained under a 12 h/12 h light/dark cycle at 25°C for 1 week. The mice were randomly divided into two groups (8 mice in each group). *H. pylori*-infected group were inoculated orally with *H. pylori* strain SS1 at a concentration of 1 × 10^8^ CFU once every other day for 4 weeks. For the control group, mice were given brain heart infusion broth instead of bacterial suspension. After another 4 weeks of feeding, all mice were sacrificed for further analysis. The animal study was reviewed and approved by the Animal Ethics Committee of Binzhou Medical University (Approval number: 2021-010).

### Statistical analyses

All results are presented as mean ± SD, and data were analyzed using GraphPad Prism software (version 8.3). Independent samples *t*-tests were used for comparing two independent samples, and one-way ANOVA for comparing multiple samples. Kaplan–Meier analysis was employed to assess survival curves, and statistical significance was set at *p* < 0.05.

## Results

### Correlation of GRB7 overexpression and *Helicobacter pylori* infection in GC tissues

**Figure 1:**
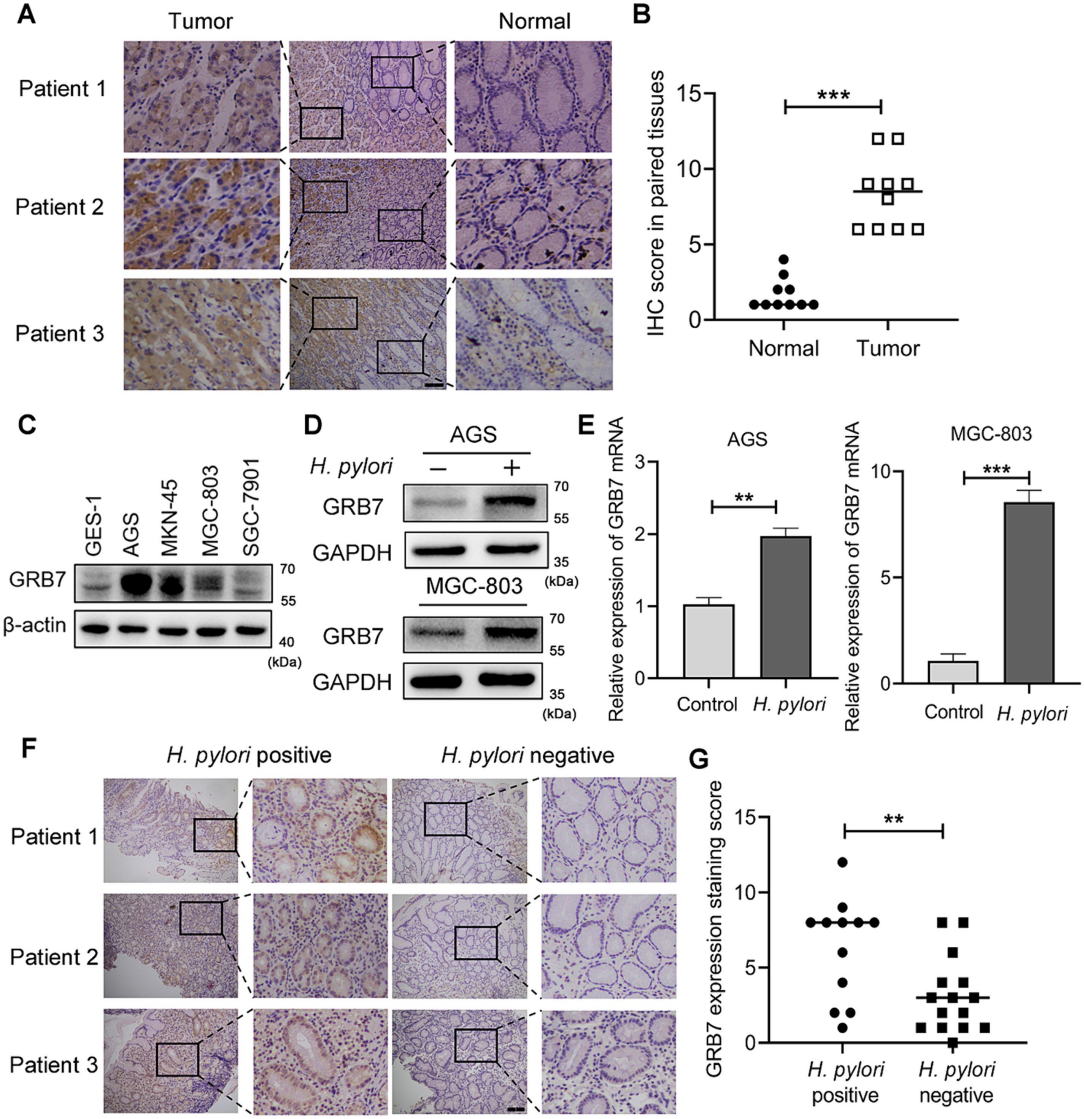
*Helicobacter pylori* increases intracellular GRB7 expression. **(A,B)** Representative IHC staining images of GRB7 in GC tissues and their paired normal gastric mucosal tissues (*n* = 10). Scale bar, 200 μm. **(B)** Quantification of **(A)**. **(C)** Expression levels of GRB7 in different cell lines. **(D,E)** Western blot **(D)** and RT-qPCR **(E)** analysis of the expression of GRB7 in cells after *H. pylori* infection. **(F,G)** Representative IHC images of GRB7 expressed in *H. pylori*-positive (*n* = 12) or -negative gastritis tissues (*n* = 15). Scale bar, 200 μm. **(G)** Quantification of **(F)**. All experiments were conducted in triplicates and the data shown are represented as mean ± SD. Data were analyzed using an independent samples *t*-test. ^**^p < 0.01 and ^***^p < 0.001.

The correlation of GRB7 expression and GC was firstly investigated. Analysis of data from GEPIA database revealed a significant upregulation of GRB7 in gastric adenocarcinoma tissues compared with that in normal gastric mucosal tissues (*p* < 0.05; Supplementary Figure S1A). Kaplan–Meier survival analysis further demonstrated that GC patients with high GRB7 expression had lower overall survival (OS) rates than those with low GRB7 expression (Supplementary Figure S1B). In order to validate these statistical data, IHC analysis of clinical samples and immunoblotting of GC cell lines were performed. From Figures 1A,B, an overexpression of GRB7 were observed in GC tissues compared with paracancer tissue. In Figure 1C, we could find that compared with epithelial cells GES-1, most GC cell lines exhibited upregulated GRB7, except for SGC-7901. Analysis of mRNA transcription level revealed a similar result, which was shown in Supplementary Figure S1C. These findings collectively substantiate the overexpression of GRB7 in GC tissues, which is consistent with previous reports (Pei et al., 2022; Zhu et al., 2023). While, the mechanism underlying this up-regulation remains unclear.

Given that *H. pylori* infection would modulate the expression of a variety intracellular molecules (Brandt et al., 2005; Sun et al., 2015; Yamaguchi et al., 2000), we investigated whether *H. pylori* influences GRB7 expression. Infection of AGS and MGC-803 cells revealed that both the protein expression and mRNA transcription levels of GRB7 significantly increased after a 4-h *H. pylori* treatment (Figures 1D,E). This examination indicated that GRB7 expression was positively associated with *H. pylori* infection. We further explored the difference in GRB7 expression between *H. pylori*-positive and -negative gastritis tissues. As illustrated by IHC representative images in Figure 1F, *H. pylori*-positive gastritis tissues exhibited higher levels of GRB7 than *H. pylori*-negative gastritis tissues (Figure 1G). These results suggested *H. pylori* infection serve as a trigger for upregulating GRB7 in gastric tissues.

### *Helicobacter pylori* induces GRB7 expression via STAT3 signaling pathway

**Figure 2:**
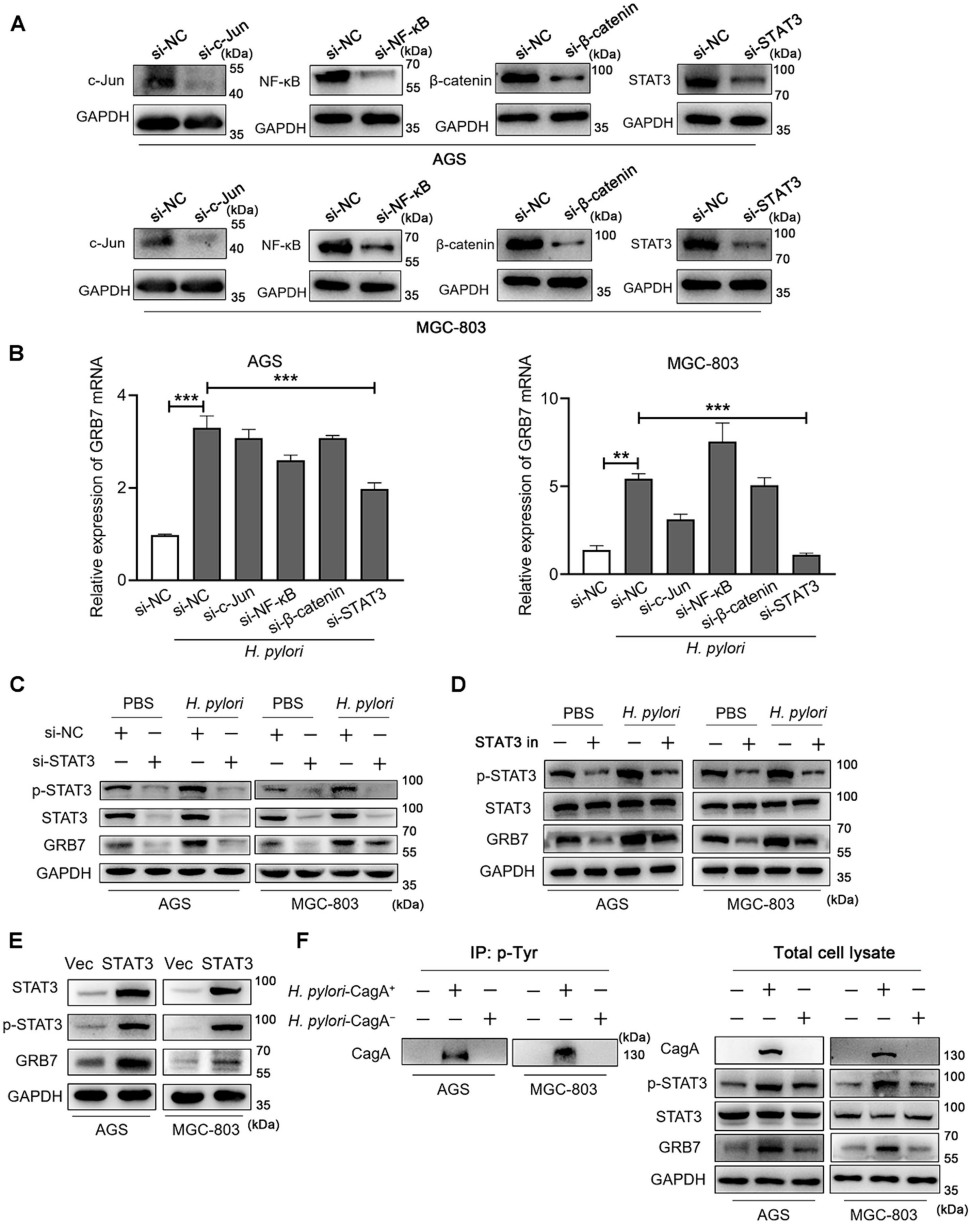
*Helicobacter pylori* induces intracellular GRB7 expression by activating STAT3 via CagA. **(A)** Western blot detection the expression of c-Jun, NF-κB, β-catenin and STAT3 with the transfection of siRNA. **(B)** The mRNA transcription level of GRB7 in cells transfected with c-Jun siRNA, NF-κB siRNA, β-catenin siRNA, STAT3 siRNA, respectively. **(C,D)** Expression levels of GRB7 and p-STAT3 in STAT3 knockdown cells **(C)** or STAT3 inhibitor-treated cells **(D)** when infected with *H. pylori*. **(E)** Expression level of GRB7 in cells overexpressing STAT3. **(F)** Expression of GRB7 and p-STAT3 in cells infected with *H. pylori* strains with or without CagA. All experiments were conducted in triplicates and the data shown are represented as mean ± SD. Statistical analysis was performed using the one-way ANOVA. si-NC indicates si-negative control. Vec indicates empty overexpressing vector pcDNA3.1. ^**^p < 0.01 and ^***^p < 0.001.

*H. pylori* is known to activate multiple transcription pathways, including the STAT3, c-Jun, NF-κB and β-catenin pathways, and then regulate the expression of intracellular genes (Franco et al., 2005; Lee et al., 2010; Bronte-Tinkew et al., 2009; Brandt et al., 2005; Sun et al., 2015). We wanted to know which pathway is involved in the regulation of GRB7. Therefore, gene knockdown experiments by using siRNAs in AGS and MGC-803 cells were performed to screen the pathway. The effectiveness of gene knockdown of STAT3, c-Jun, NF-κB, and β-catenin was detected by western blot assays and the results are shown in Figure 2A. Focus on the expression of *GRB7*, we found *STAT3* knockdown dramatically reduced the upregulation of *GRB7* mRNA induced by *H. pylori* (Figure 2B). Western blot results also demonstrated that a reduction in the intracellular STAT3 level led to a corresponding decrease in GRB7 expression (Figure 2C). In contrast, knockdown of other molecules had no steady effect. The inhibitor (Stattic) was also used to inhibit the phosphorylation of STAT3, and the decreased in GRB7 expression was also observed (Figure 2D), which confirmed that the regulation of GRB7 expression was mediated by STAT3 activation. To further confirm this relationship, we overexpressed STAT3 by transfecting recombinant pcDNA3.1-STAT3 plasmids into AGS and MGC-803 cells. This manipulation was proved to result in increased GRB7 expression (Figure 2E). These findings indicate that *H. pylori*-induced GRB7 upregulation may depend on STAT3 activation.

STAT3 is a well-established transcription factor implicated in oncogenesis and plays a pivotal role in GC development (Bronte-Tinkew et al., 2009; Yamaguchi et al., 2000). CagA, the key virulence factor of *H. pylori*, was reported to have the ability to activate STAT3 signaling pathway, thereby promoting GC development. We speculated that CagA is functional in STAT3-associated overexpression of GRB7 during *H. pylori* infection. To verify this, we compared GRB7 expression levels following infection with CagA positive *H. pylori* strain (*H. pylori*-CagA^+^) and CagA negative strain (*H. pylori*-CagA^−^) at the same infection multiplicity. The tyrosine phosphorylated CagA was detected in the cells infected with CagA positive *H. pylori*, suggesting the injection of CagA (Figure 2F). We found the upregulation of GRB7 was less pronounced in *H. pylori*-CagA^−^ infected cells compared with that in CagA positive infection (Figure 2F), suggesting that CagA is involved in the process of *H. pylori*-induced GRB7 overexpression.

### *Helicobacter pylori* disrupts proliferation-apoptosis balance through GRB7

**Figure 3:**
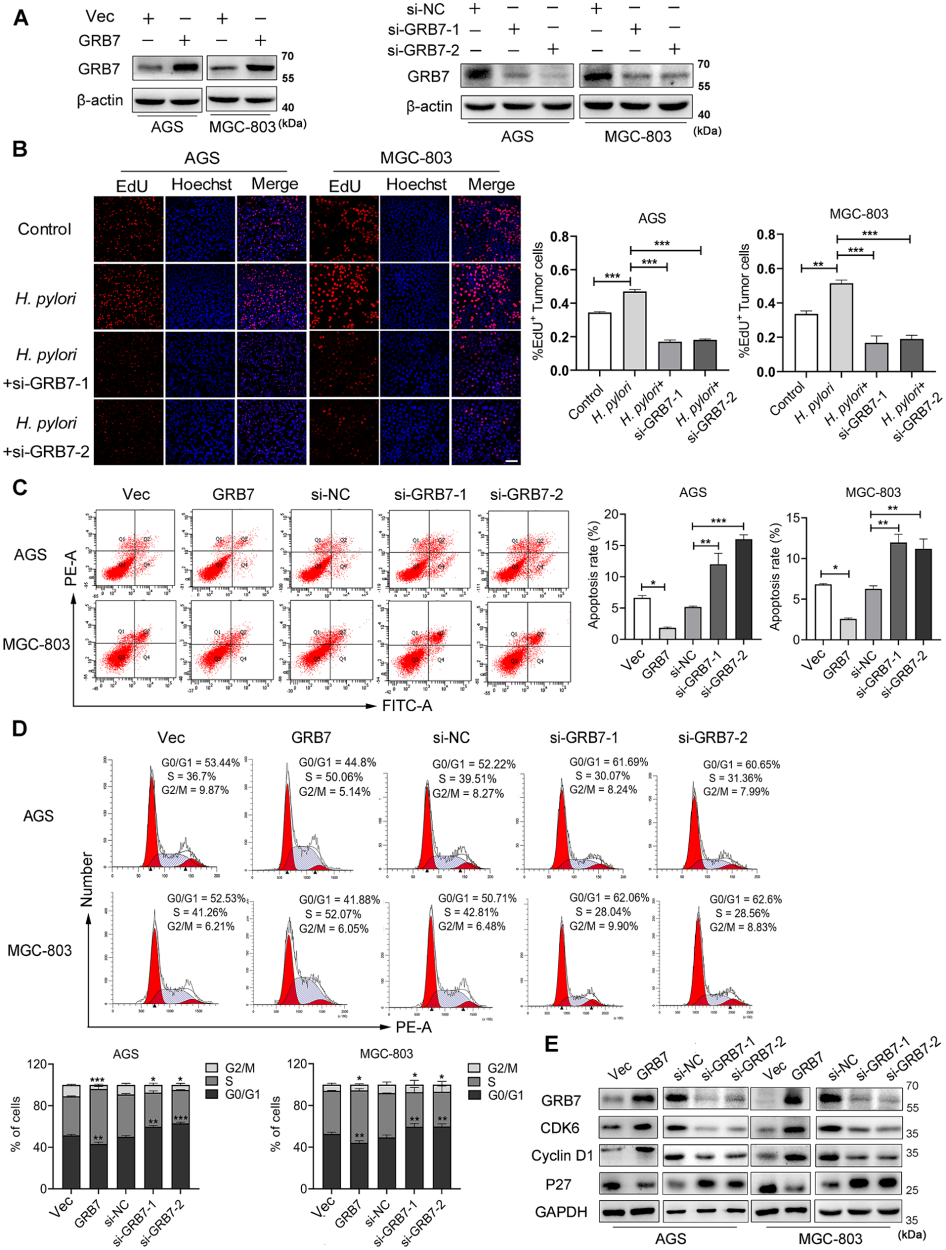
*Helicobacter pylori* promotes cell proliferation via GRB7. **(A)** Western blot analysis of GRB7 knockdown and overexpression efficiency in cells. **(B)** EdU analysis of the proliferative capacity of cells. Scale bar, 60 μm. **(C,D)** The flow cytometry determination of the apoptosis rate **(C)** and percentage of cells in different cell cycle phases **(D)**. **(E)** Western blotting detection of the expression of cycle-critical proteins. All experiments were conducted in triplicates and the data shown are represented as mean ± SD. Statistical analysis was performed using the one-way ANOVA. ^*^p < 0.05, ^**^p < 0.01, and ^***^p < 0.001.

*H. pylori*-induced proliferation on cells damages the integrity of gastric mucosal epithelium, even promotes tumorigenesis (Yamaguchi et al., 2000; Nadler et al., 2010). GRB7 has been reported to be implicated in the proliferation of cervical and ovarian cancers, which inspires us to investigate whether the upregulated GRB7 promotes the proliferation of GC cells during *H. pylori* infection. GRB7 knockdown and overexpression were conducted by transfecting cells with GRB7 siRNA or pcDNA3.1-GRB7, respectively (Figure 3A), and assessed cell proliferation using EdU staining as a marker. The results revealed that approximately 30% of normal cells incorporated EdU, indicating active cell proliferation, and *H. pylori* infection significantly enhanced this ratio. Conversely, the number of EdU-positive cells substantially decreased in GRB7 knockdown mutant cells (Figure 3B). This result supports the hypothesis that GRB7 participates in *H. pylori*-stimulated cell proliferation.

Next, how excess GRB7 improves cell proliferation was explored. So GRB7 overexpression cell mutant was employed to investigate this problem. Both colony formation assay and EdU experiment confirmed the role of GRB7 in affecting cell proliferation (Supplementary Figures S2A,B). Since our body normally maintains its homeostasis of cell number through the balance of proliferation and apoptosis, the effect of GRB7 on cell’s apoptosis was examined. Flow cytometry showed that the percentage of apoptotic cells was lower in GRB7-overexpressed cells than in the control, whereas it was higher in GRB7-silenced cells (Figure 3C). These results confirmed the recent reports (Zhao et al., 2017; Chu et al., 2010) and indicates that overexpressed GRB7 could disturb the balance of proliferation-apoptosis by decreasing the apoptosis and enhancing the proliferation of cells. As the cell cycle is closely linked to cell proliferation and apoptosis, we further analyzed the effect of GRB7 on the cell cycle. As shown in Figure 3D, GRB7 overexpression significantly increased the proportion of cells in S phase and reduced cells in G0/G1 phase. Conversely, silencing GRB7 led to the opposite effect: a marked decrease in the proportion of cells in the S phase accompanied by an increase in G0/G1 phase. Detection of several key factors regulating the cell cycle also indicated the effect of GRB7 on cell cycle transition (Figure 3E). We can find that the cell cycle promoters, cyclin D1 and CDK6, were upregulated in GRB7-expressing cells and down-regulated in GRB7-silencing cells. Moreover, the cell cycle inhibitor p27 was decreased in GRB7-overexpressed cells and elevated in GRB7-silenced cells. These results indicate that GRB7 enhances the proliferation through promoting the cell cycle G1/S transition in GC cells.

Based on these findings, it could be inferred that *H. pylori* regulates the cell cycle by upregulating GRB7, thus disrupting the equilibrium between proliferation and apoptosis in gastric cells.

### GRB7 is involved in *Helicobacter pylori*-induced cell migration and invasion

**Figure 4:**
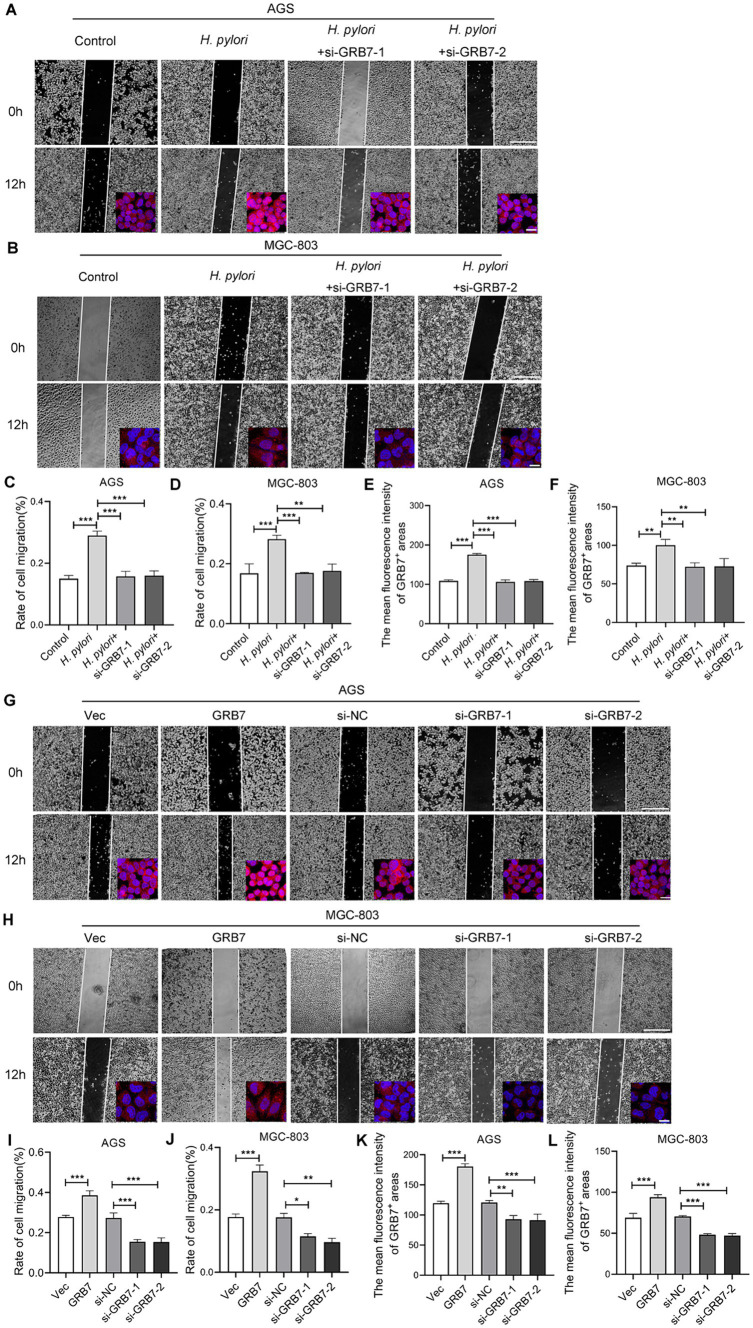
Analysis the effect of GRB7 on cell migration by wound-healing assays. **(A,B)** Detection the migration of AGS and MGC803 cells with *H. pylori* infection and GRB7 knockdown. The expression of GRB7 was detected by immunofluorescence and results showed in bottom right corner of each image. **(C,D)** Quantification results of cell migration. **(E,F)** Fluorescence statistics. **(G,H)** Measurement the migration of AGS and MGC803 cells with GRB7 overexpression or knockdown. Immunofluorescence images of GRB7 expression were also shown in the lower right. **(I,J)** Quantification results of cell migration. **(K,L)** Fluorescence statistics. Scale bar for representative images of wound healing assay, 500 μm. Scale bar for representative images of immunofluorescence, 20 μm. All experiments were conducted in triplicates and the data shown are represented as mean ± SD. Statistical analysis was performed using the one-way ANOVA. ^*^p < 0.05, ^**^p < 0.01, and ^***^p < 0.001.

Alterations in cell motility are widely recognized as a crucial factor in the initiation and progression of tumor metastasis. *H. pylori* inducing cell migration to facilitate GC metastasis has been widely reported (Amieva and Peek, 2016; Wu et al., 2013; Yang et al., 2018; Sun et al., 2015; Lee et al., 2012). We then detected the impact of GRB7 on cell migration during *H. pylori* infection. Transwell and wound healing assays were both used to detect cell motility. As shown in Figures 4A–D, the wound-healing ability in cells increased under *H. pylori* infection. However, after knocking down GRB7, the increase caused by the infection disappeared. The expression of GRB7 was detected by immunofluorescence, and the results are presented in the bottom right corner of each image. The brighter fluorescence of the cells was observed due to infection with *H. pylori*, suggesting upregulated expression of GRB7 (Figures 4E,F). When GRB7 was overexpressed and knocked down in cells, the results showed that the wound healing ability of cells was positively correlated with the expression level of GRB7 (Figures 4G–J). The immunofluorescence results of the cells demonstrated the efficacy of both GRB7 overexpression and knockdown. Reduced fluorescence intensity of GRB7 indicates decreased protein expression following siRNA inhibition, whereas enhanced fluorescence is observed upon overexpression of GRB7 using transfected plasmids (Figures 4K,L).

**Figure 5:**
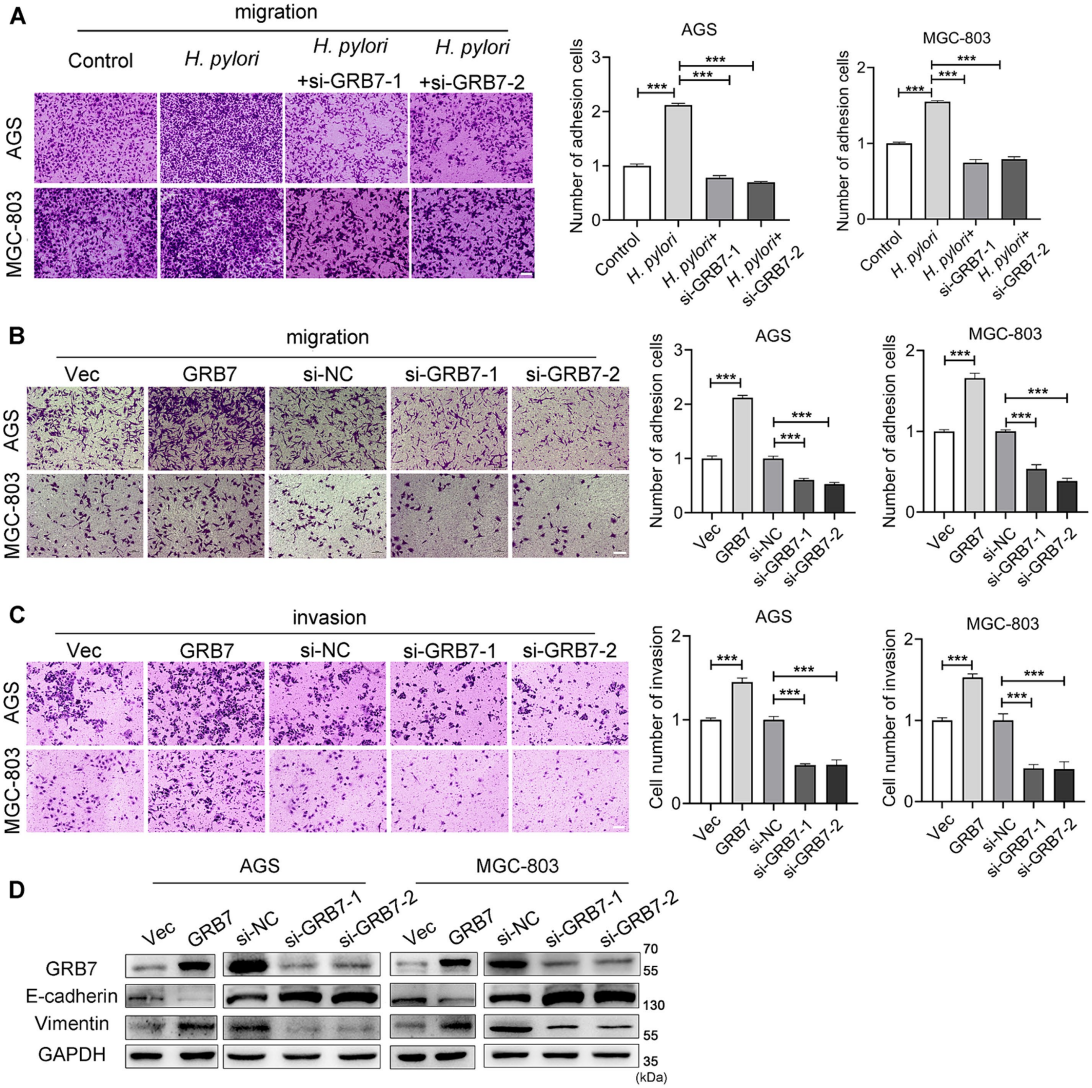
Analysis the effect of GRB7 on cell motility by Transwell assays. **(A)** Knockdown of GRB7 affects *H. pylori* inducing cells migration. **(B,C)** Role determination of GRB7 in cell migration and invasion. **(D)** Expression of EMT markers in cells knockdown or overexpression of GRB7. Scale bar, 100 μm. All experiments were conducted in triplicates and the data shown are represented as mean ± SD. Statistical analysis was performed using the one-way ANOVA. ^***^p < 0.001.

The Transwell data also demonstrated that knockdown of GRB7 expression inhibited *H. pylori*-induced migration (Figure 5A). When we overexpressed GRB7 in cells to simulate the effect of *H. pylori* infection, cell migration was significantly enhanced (Figure 5B). Cell invasion assays showed the same trend with the migration (Figure 5C). This suggests that GRB7 is involved in the progress of cell migration and invasion induced by *H. pylori*.

Given the critical role of EMT in tumor invasion and metastasis, we assessed the effect of GRB7 on EMT. As shown in Figure 5D, GRB7 overexpression resulted in decreased expression of the epithelial cell marker E-cadherin and increased expression of the mesenchymal cell marker vimentin, whereas GRB7 knockdown exhibited the opposite effects.

These results implied that GRB7 participates in *H. pylori*-induced cell motility via regulating EMT, which in turn promotes GC metastasis.

### Activation of ERK pathway by GRB7 contributes to promoting neoplastic traits by *Helicobacter pylori*

**Figure 6:**
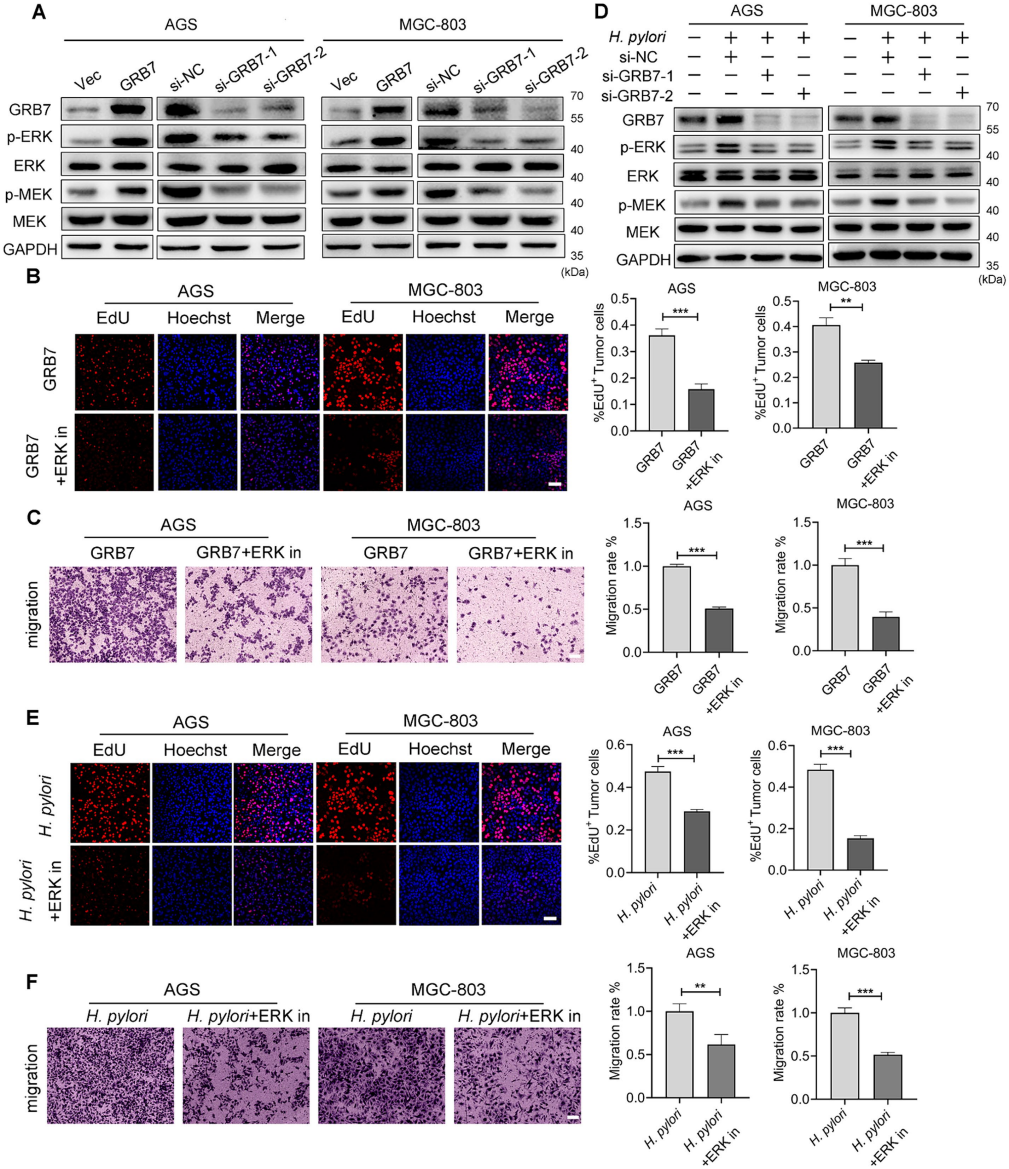
*Helicobacter pylori* regulates cell biology by GRB7-ERK pathway. **(A)** Western blot examination for ERK activation by GRB7. **(B)** EdU assay for proliferative capacity of cells when overexpressing GRB7 and treated with ERK inhibitor. **(C)** Determination of the migratory capacity of cells when overexpressing GRB7 and treated with ERK inhibitor. **(D)** Western blot detection of the association between GRB7 and ERK pathway activation during *H. pylori* infection. **(E)** EdU assay indicating the proliferative capacity of cells under *H. pylori* infection and ERK inhibitor treatment. Scale bar, 60 μm. **(F)** Migration of cells under *H. pylori* infection and ERK inhibitors treatment. Scale bar, 100 μm. All experiments were conducted in triplicates and the data shown are represented as mean ± SD. Data were analyzed using an independent samples *t*-test. ^**^p < 0.01, and ^***^p < 0.001.

The aforementioned studies have shown that overexpressed GRB7 can regulate cell cycle and EMT, causing abnormal proliferation and motility, thereby enhancing the neoplastic traits of cells. Next, we intended to explore the bridge between GRB7 and these phenotypes. The ERK signaling is one of the important pathways associated with proliferation and migration in various cancers (Roskoski, 2017; Chen et al., 2021; Savoia et al., 2019; Zhang et al., 2022), through western blot assay, we found that the levels of p-ERK in AGS and MGC-803 cells were significantly increased by GRB7 overexpression (Figure 6A). Moreover, the phosphorylation level of MEK, a key molecule upstream of ERK, exhibited a similar change. But when we added ERK inhibitor into cell culture, the increased proliferating cells and migrated cells promoted by GRB7 overexpression reduced to a normal level (Figures 6B,C). These findings suggested that MEK/ERK signaling is an important downstream pathway through which GRB7 plays roles.

During *H. pylori* infection, the phosphorylation of ERK and MEK were also increased. While, silencing GRB7 could reverse these changes (Figure 6D). The addition of an ERK inhibitor can effectively reduce the proliferation and migration caused by *H. pylori* (Figures 6E,F). These results highlighted the role of GRB7-ERK pathway in *H. pylori* infection.

### Confirmation of STAT3-GRB7-ERK cascade in *Helicobacter pylori* infected mice model

**Figure 7:**
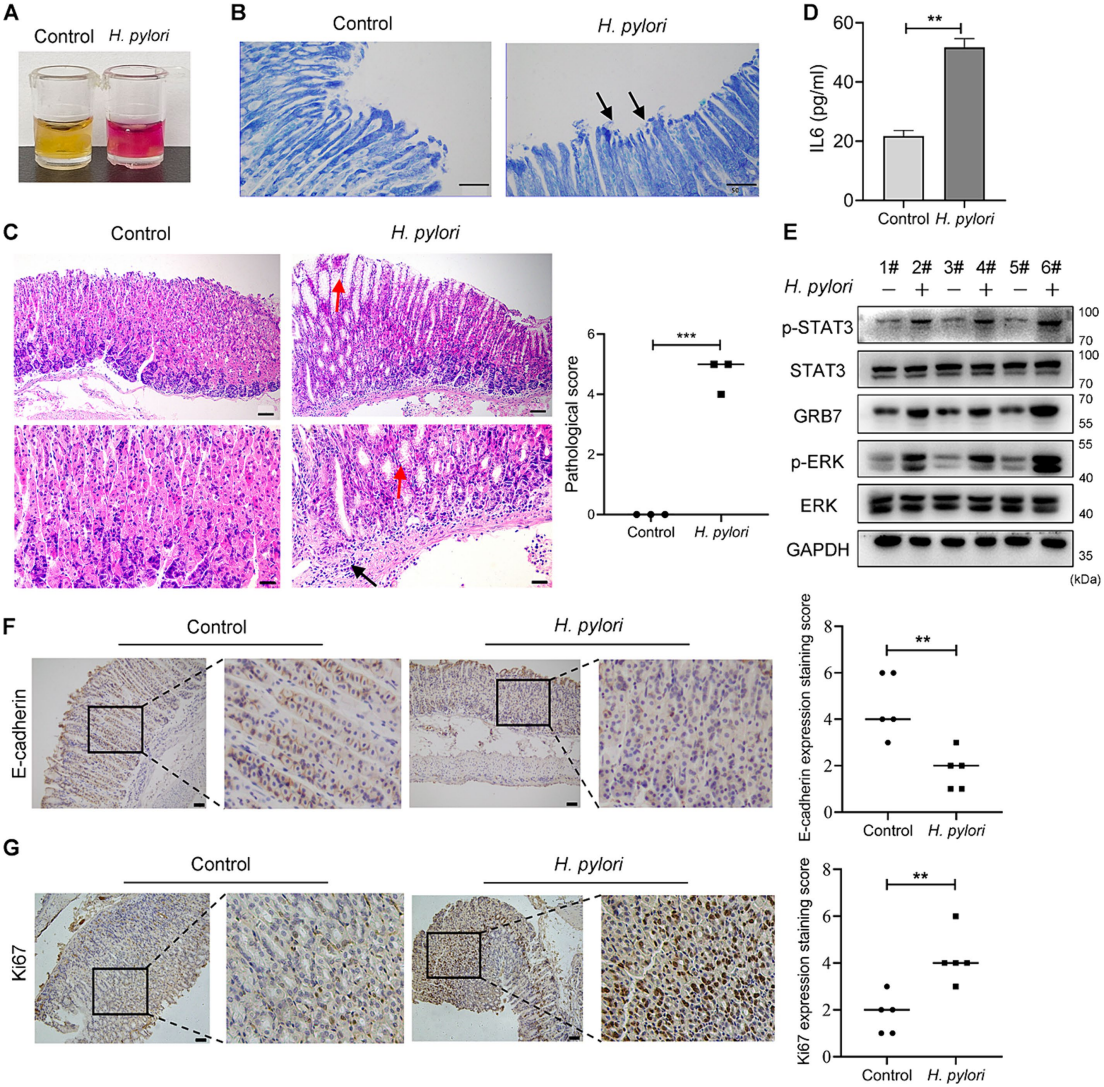
Confirmation of GRB7 roles in *H. pylori* pathogenesis *in vivo*. **(A,B)** Rapid urease test **(A)** and Giemsa staining **(B)** confirming *H. pylori* colonization. Black arrows indicate curved *H. pylori* (400×). **(C)** HE staining of mice gastric epithelium. Red arrows indicate rounded vacuolated cup-shaped cells, black arrows show inflammatory cells (*n* = 3). Bar in upper image indicates 100 μm, and in lower image indicates 50 μm. **(D)** The amount of IL6 in serum. **(E)** Expression of GRB7, p-ERK, and p-STAT3 in gastric tissues of mice. **(F,G)** Expression of E-cadherin and Ki67 in gastric mucosa detected by IHC (*n* = 5). Scale bar, 100 μm. All experiments were conducted in triplicates and the data shown are represented as mean ± SD. Data were analyzed using an independent samples *t*-test. Control, brain heart infusion broth treated mice; *H. pylori*, *H. pylori*-infected mice. ^**^p < 0.01, and ^***^p < 0.001.

**Figure 8:**
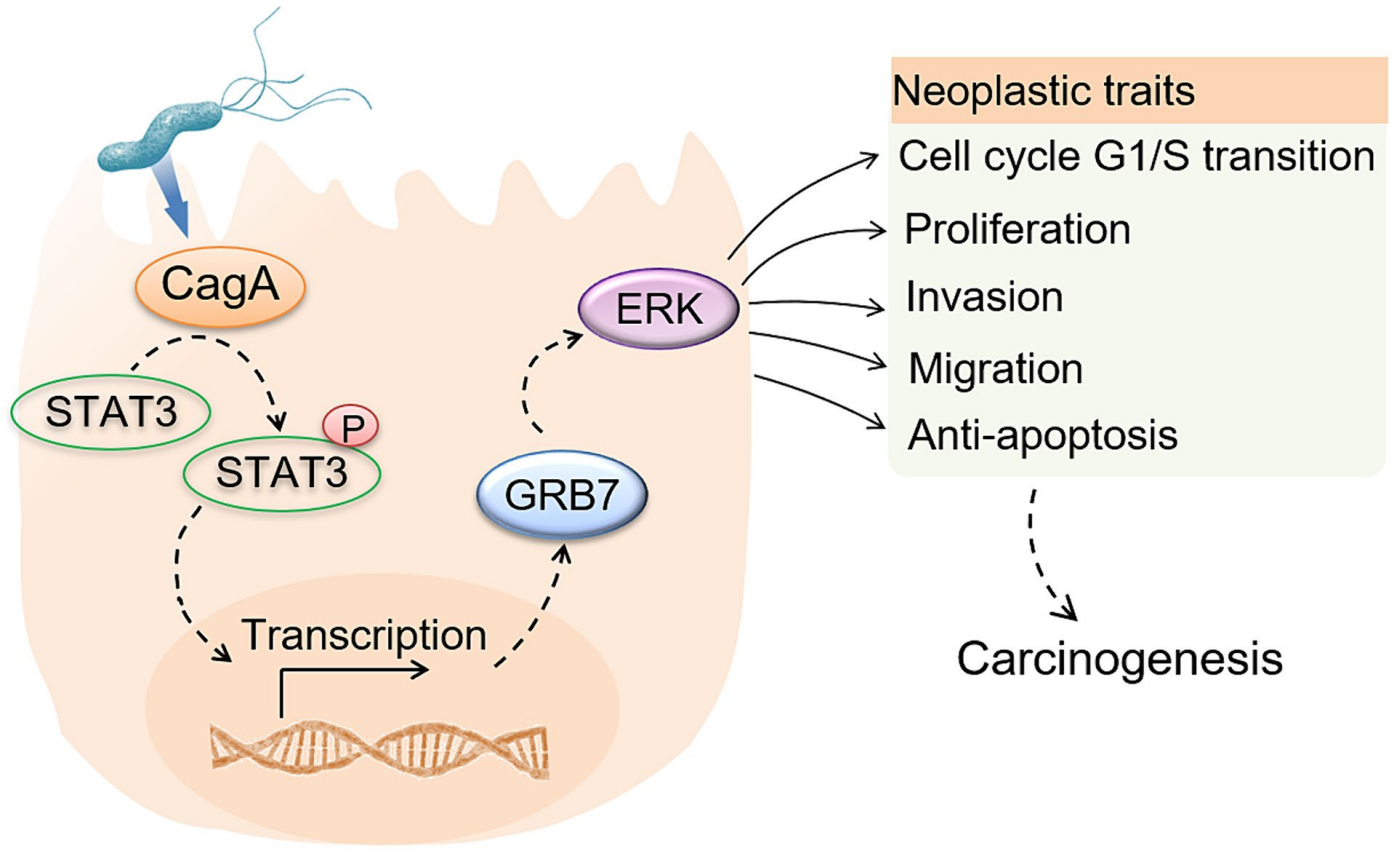
The illustrated graph of GRB7-mediated *H. pylori* pathogenesis.

To confirmation of the action of GRB7 in *H. pylori* infection *in vivo*, mice infection assays were carried out. *H. pylori* colonization was confirmed by both rapid urease test and Giemsa staining (Figures 7A,B). Figure 7C showed the pathological analysis results. We can see that in control, the structure of the gastric mucosal layer was clear, and the tissue did not show inflammatory cell infiltration. In *H. pylori* infected gastric mucosa, pathological changes including partial enterozation of the mucosal layer, rounded vacuolar cup-shaped cell proliferation and infiltration of inflammatory cells at the bottom of the mucosal layer were observed. Higher IL6 secretion was also detected in the serum of infected mice, revealing a *H. pylori*-induced inflammation (Figure 7D). As expected, higher expression of GRB7 was observed in infected gastric tissues (Figure 7E). More p-STAT3 and p-ERK were also detected, indicating the abnormal activation of STAT3 and ERK pathway (Figure 7E). IHC staining exhibited higher expression levels of Ki67 and E-cadherin in *H. pylori* infected tissues, which reflected the proliferative and metastatic effect induced by *H. pylori* infection (Figures 7F,G). These data partially supported our idea about GRB7 in *H. pylori*-induced gastric disorders, although the causality between these results remains to be clarified further (see Figure 8).

## Discussion

Gene *GRB7*, which encodes 535 amino acids, is located on chromosome 17q12 along with *ErbB2*. It initially garnered attention due to its frequent co-amplification with *ErbB2* in various human cancers (Selbach et al., 2009; Meric-Bernstam et al., 2019; Varis et al., 2002; Lim et al., 2014). Upregulation of GRB7 has been observed in breast cancer, bladder cancer, ovarian cancer, esophageal cancer, and GC (Wang et al., 2010; Zheng et al., 2020; Gotovac et al., 2020; Pei et al., 2022; Nadler et al., 2010). Clinical data from various cases reveal that GRB7 expression level is often negatively correlated with patient prognosis (Zheng et al., 2020; Gotovac et al., 2020; Selbach et al., 2009; Nadler et al., 2010), suggesting a noteworthy link between GRB7 and cancer development. The impact of GRB7 on tumor progression has been investigated in several studies, with consistent findings indicating its enhancement of cell proliferative, migratory, and invasive properties. Inhibition of GRB7 mitigates these oncogenic transformations (Zheng et al., 2020; Wang et al., 2020; Zhao et al., 2017; Pei et al., 2022). These studies not only imply an oncogenic role in tumorigenesis for this protein, but also suggest the potential for targeting GRB7 to inhibit carcinogenesis.

The understanding of GRB7 action in GC is still incomprehensive now. Earlier researches have confirmed the high transcription of *GRB7* in human GC (Pei et al., 2022; Meric-Bernstam et al., 2019; Varis et al., 2002). Recently, this high transcription is verified on the expression level, which was also found to negatively correlate with patient prognosis (Pei et al., 2022; Zhu et al., 2023) and contribute to the proliferation and migration of GC cells (Zhu et al., 2023). In this study, we also confirmed its upregulation in GC tissues by both database and clinical analysis. The effects of upregulated GRB7 in human GC cells were also identified, and found that overexpressed GRB7 reduced cell apoptosis and enhanced cell proliferation, migration and invasion. These results strongly implicate an active participation of GRB7 in GC development.

As a typical adaptor protein, GRB7 participates in the transduction of multiple signal pathways (Han et al., 2001; Chu et al., 2019; Paudyal et al., 2013; Pero et al., 2003). The SH2 domain in its C-terminal plays a critical role in the signal transduction cascades. It achieves this by binding to activated receptor tyrosine kinases/nonreceptor tyrosine kinases or other tyrosine-phosphorylated signaling proteins. These proteins include EGFR, ERBB2, ERBB3, ERBB4, Ret, EphB1, c-Kit, FAK, Tek/Tie2, and SHPTP2 (Thommes et al., 1999; Janes et al., 1997; Keegan and Cooper, 1996; Fiddes et al., 1998; Pandey et al., 1996; Han et al., 2002; Jones et al., 1999). The interaction with these molecules recruits and activates GRB7, enabling it to engage with downstream partners that mediate multiple cell signal transduction pathways, such as ERK, Ras, and Akt pathways (Wang et al., 2010; Zheng et al., 2020; Chu et al., 2010). These activation leads to distinct cellular outcomes or tumorigenesis. In our study, we discovered a significant relationship between the expression of GRB7 and ERK activation, both of which were jointly correlated with GC progression. Utilizing inhibitors, we characterized the role of GRB7/ERK signaling in promoting GC cell migration and invasion. These findings align with those research in ovarian cancer (Wang et al., 2010; Han and Guan, 1999). Moreover, Itoh et al. (2007) reported the involvement of GRB7 in hepatocellular carcinoma progression associated with the expression of FAK. Zheng et al. (2020) found that GRB7 promoted G1/S transition by activating the AKT pathway in bladder cancer. Further research is needed to elucidate whether GRB7 also regulates other signalings in gastric carcinogenesis. Nevertheless, GRB7 and its downstream ERK pathway appear to be promising therapeutic targets in GC.

GC continues to rank among the most lethal neoplasms globally, presenting a challenge due to limited therapeutic options. Given the suboptimal efficacy of targeted therapies, there is a pressing need for the target identification. Extensive studies have been devoted in this region, and several potential oncogenic molecules that regulate the progression of GC have been reported. Fibroblast Growth Factor Receptor 2 (FGFR2) was regarded as a promising target for GC, and there has been an increasing number of small molecule inhibitors and monoclonal antibodies targeting FGFR2 that have entered into clinical trials (Tojjari et al., 2024). Gankyrin was found to contribute to the early malignant transformation of GC and can be selected to predict the risk of GC in those patients harboring the precancerous lesions (dysplasia and intestinal metaplasia) (Huang et al., 2018). Several preclinical studies have identified histone deacetylases (HDAC) as potential therapeutic targets in GC (Jenke et al., 2024). In recent studies, NUF2 was also reported to be a potential therapeutic target to inhibit GC progression (Long et al., 2024). UBR1 was found to be a prognostic biomarker and therapeutic target associated with immune cell infiltration in GC (Yuan et al., 2024). These target’s explorations provide more options for the development of pharmacological agents that operate GC inhibition through broader mechanisms.

*Helicobacter pylori* is recognized as the primary etiological agent responsible for the development of GC (Amieva and Peek, 2016; Wroblewski and Peek, 2023; Wu et al., 2013; Cover and Blaser, 2009; Yang et al., 2018). It can dysregulate host intracellular signaling pathways and lower the threshold for neoplastic transformation (Hatakeyama, 2014; Asahi et al., 2000; Higashi et al., 2002). In our study, we observed an increase in GRB7 expression following *H. pylori* infection in both *in vitro* and in clinical samples. Given the carcinogenic potential of GRB7, we identified a pathogenic pathway of *H. pylori* in which upregulated GRB7 activates ERK signaling, promoting neoplastic transformation by enhancing cell proliferation, migration, and invasion. Subsequently, we investigated how GRB7 is activated by bacterial infection. Based on siRNA silencing assay, JAK2/STAT3 gene regulatory signaling was screened out to be involved in this progress. As reported, *H. pylori* could active JAK2/STAT3 signaling through its CagA, a virulence factor of *H. pylori* to promote cell migration and invasion (Bronte-Tinkew et al., 2009; Sun et al., 2015; Lee et al., 2012). Further investigation was performed to extend our knowledge about GRB7 expressing, which indicated that activation of STAT3 by *H. pylori* CagA is mainly responsible for promoting GRB7 expression in GC cells. While, the injection of CagA into host cells requires a complete type IV secretory system. So, we believe that interference translocation of CagA would also affect the activation of STAT3 and thus the expression of GRB7. For example, the *cagL* gene is involved in the translocation of CagA (Conradi et al., 2012), might also indirectly involve in the regulation of GRB7 by *H. pylori*.

CagA is a particularly significant virulence factor of *H. pylori* as it is closely associated with the development of GC (Hatakeyama, 2014; Tojjari et al., 2024; Huang et al., 2018; Jenke et al., 2024; Long et al., 2024; Yuan et al., 2024; Asahi et al., 2000; Higashi et al., 2002; Conradi et al., 2012; Fajardo et al., 2013). During infection, CagA is injected into host cells through the type IV secretory system (T4SS) and serves as a docking site for intracellular interactions (Asahi et al., 2000; Higashi et al., 2002; Conradi et al., 2012; Fajardo et al., 2013). The glutamate-proline-isoleucine-tyrosine-alanine (EPIYA) motif on its C-terminal can be phosphorylated on its tyrosine residues by host tyrosine kinases of Src and Abl families, enabling it to interact with various SH2-containing proteins, such as GRB2, SHP1, SHP2, Csk, SHIP2 (Higashi et al., 2002; Fajardo et al., 2013). Given the SH2-containing, GRB7 was speculated to interact with CagA directly, and this interaction was firstly reported by Selbach et al. (2009) through high-resolution mass spectrometry and Co-IP assay. Whether CagA regulates the action of GRB7 directly in addition to via STAT3 warrants further investigation.

Based on results in this study, we provided a hypothesis that *H. pylori* infection upregulates GRB7 expression by CagA mediated STAT3 activation, further activates ERK signaling, promoting cell proliferation by cell cycle regulation and motility by EMT induction. Our results support the promoting effect of GRB7 in GC development, and highlight GRB7-mediated enhancement of cell malignant phenotypes in *H. pylori* infection, which offer new prospects for the treatment and prevention of *H. pylori*-associated gastric carcinogenesis.

It should be noted that *H. pylori* strains have a high diversity in genomic DNA nucleotide sequences. This genetic diversity would affect the function and antigenicity of virulence factors associated with bacterial infection and, ultimately, disease outcome (Han et al., 1998). Due to strain limitations, our results in this study cannot be representative of all *H. pylori* infections.

## Conclusion

Our findings demonstrate a novel pathogenic pathway associated with *H. pylori* infection. *H. pylori* activates STAT3, leading to increased GRB7 expression. This, in turn, promotes the activation of ERK pathway, resulting in increased cell proliferation, migration and invasion. Our results elucidate the role of GRB7 in *H. pylori*-induced gastric disorders and offer new prospects for treating and preventing *H. pylori*-associated gastric carcinogenesis.

## Data Availability

The original contributions presented in the study are included in the article/Supplementary material, further inquiries can be directed to the corresponding author.
