# Supplementary material for: GRB7-mediated enhancement of cell malignant characteristics induced by Helicobacter pylori infection
Source: Front Microbiol. 2024 Sep 18;15:1469953. doi: 10.3389/fmicb.2024.1469953 (PMC11444978; doi:10.3389/fmicb.2024.1469953)
Supplement: Supplementary file 1 [file Data_Sheet_1.PDF]

Supplementary Material

Supplementary Table

Supplementary Table 1. The sequences of siRNA used in the study

Name	Sequences	Reference(s) or source
si-GRB7-1	CGCCAAGUACGAACUGUUCAA	This study
si-GRB7-2	CCAGGGCUUUGUCCUCUCUUU	This study
si-STAT3	GCAAGAUUCAGACCCUCAATT	1
si-NF-κB	GGACAUAUGAGACCUUCAA	2
si-c-Jun	AGAUGGAAACGACCUUCUA	3
si-β-catenin	ACAUCGAAGACUCUACAAU	4

Supplementary Figures

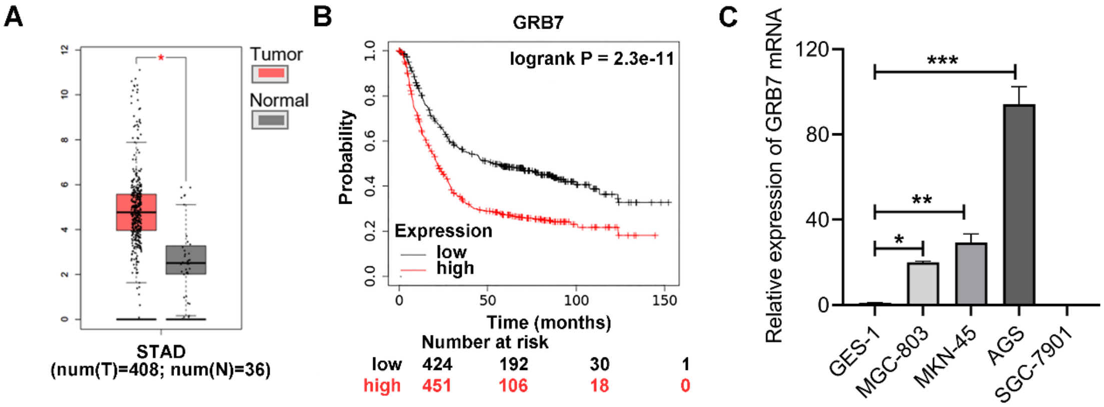

Supplementary Figure 1. GRB7 is up-regulated in gastric cancer tissues and cells. (A) The mRNA level analysis of GRB7 in normal gastric mucosa tissue (n=36) and gastric adenocarcinoma tissue (n=408) based on GEPIA database. (B) Kaplan–Meier OS analysis of the relationship between GRB7 expression and the patients' prognosis (low expression n=424, high expression n=451). (C) The mRNA expression of GRB7 detected by RT-qPCR in cell lines. The experiment was conducted in triplicates and the data shown are represented as mean ± SD. Statistical analysis was performed using the one-way ANOVA. *P < 0.05, **P < 0.01, ***P < 0.001.

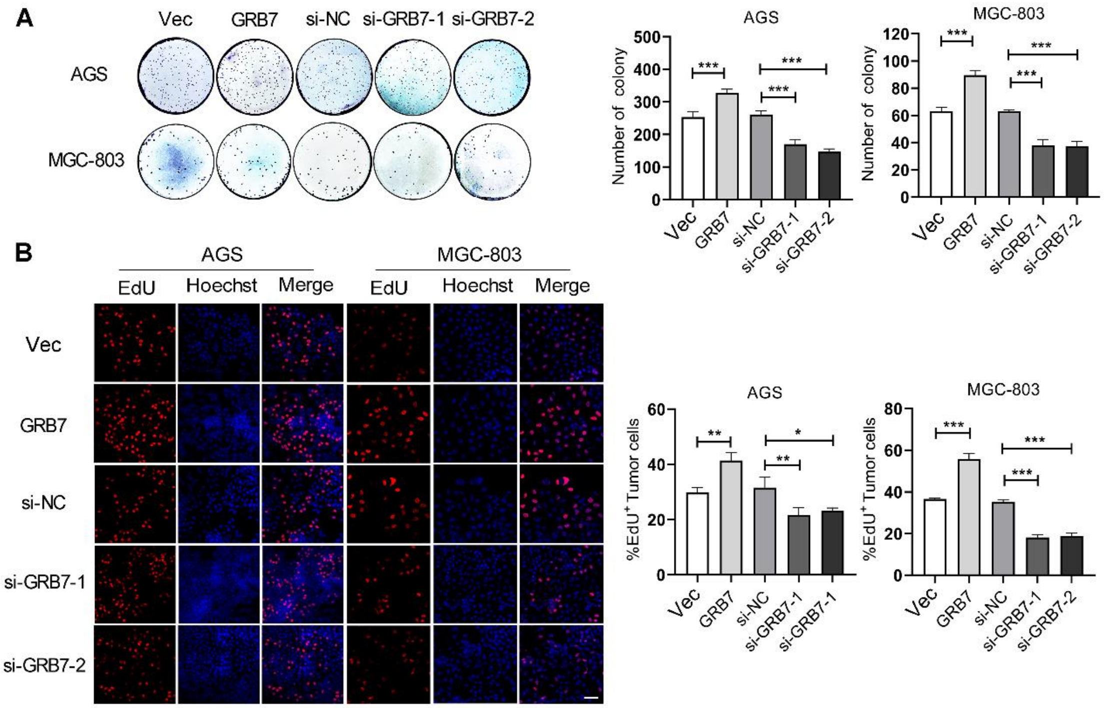

Supplementary Figure 2. The effect of GRB7 on cell proliferation. (A) Colony formation assays indicating the proliferation of cells knocking down or overexpressing GRB7. (B) EdU shows the percentage of cells in the DNA synthesis phase after GRB7 overexpression and knockdown. Scale bar, 60 μm. All experiments were conducted in triplicates and the data shown are represented as mean ± SD. Statistical analysis was performed using the one-way ANOVA. *$P < 0.05$, **$P < 0.01$, ***$P < 0.001$.

References:

1. Yuan, W., Li, T., Mo, X., Wang, X., Liu, B., Wang, W., et al. (2016). Knockdown of CMTM3 promotes metastasis of gastric cancer via the STAT3/Twist1/EMT signaling pathway. *Oncotarget* 7(20), 29507-29519. doi: 10.18632/oncotarget.8789.

2. Jeong, J.Y., Woo, J.H., Kim, Y.S., Choi, S., Lee, S.O., Kil, S.R., et al. (2010). Nuclear factor-kappa B inhibition reduces markedly cell proliferation in Epstein-Barr virus-infected stomach cancer, but affects variably in Epstein-Barr virus-negative stomach cancer. *Cancer Invest* 28(2), 113-119. doi: 10.3109/07357900903095730.

3. Li, D.D., Wang, L.L., Deng, R., Tang, J., Shen, Y., Guo, J.F., et al. (2009). The pivotal role of c-Jun NH2-terminal kinase-mediated Beclin 1 expression during anticancer agents-induced autophagy in cancer cells. *Oncogene* 28(6), 886-898. doi: 10.1038/onc.2008.441.

4. Chen, X.Y., Wan, S.F., Yao, N.N., Lin, Z.J., Mao, Y.G., Yu, X.H., et al. (2021). Inhibition of the immunoproteasome LMP2 ameliorates ischemia/hypoxia-induced blood-brain barrier injury through the Wnt/beta-catenin signalling pathway. *Mil Med Res* 8(1), 62. doi: 10.1186/s40779-021-00356-x.
